# Mobilizing Endogenous Repair Through Understanding Immune Reaction With Biomaterials

**DOI:** 10.3389/fbioe.2021.730938

**Published:** 2021-11-30

**Authors:** Maria Karkanitsa, Parinaz Fathi, Tran Ngo, Kaitlyn Sadtler

**Affiliations:** Section on Immuno-Engineering, National Institute of Biomedical Imaging and Bioengineering, National Institutes of Health, Bethesda, MD, United States

**Keywords:** biomaterials, immunoengineering, regenerative medicine, wound healing, hydrogels, foreign body response, skin regeneration, muscle regeneration

## Abstract

With few exceptions, humans are incapable of fully recovering from severe physical trauma. Due to these limitations, the field of regenerative medicine seeks to find clinically viable ways to repair permanently damaged tissue. There are two main approaches to regenerative medicine: promoting endogenous repair of the wound, or transplanting a material to replace the injured tissue. In recent years, these two methods have fused with the development of biomaterials that act as a scaffold and mobilize the body’s natural healing capabilities. This process involves not only promoting stem cell behavior, but by also inducing activity of the immune system. Through understanding the immune interactions with biomaterials, we can understand how the immune system participates in regeneration and wound healing. In this review, we will focus on biomaterials that promote endogenous tissue repair, with discussion on their interactions with the immune system.

## Introduction

Biomaterial properties such as mechanics, chemical composition, biodegradability, and others play a role in governing biomaterial-tissue interactions. Despite early attempts, it was quickly understood that an immune response to the biomaterial could not be avoided (Williams, 2008). The classical negative immune response to a biomaterial is termed the Foreign Body Response (FBR), which involves protecting the body from the invading material *via* a fibrous capsule around the material (Zhang et al., 2021). Recent efforts have focused on developing biomaterials that actively promote a regenerative environment and healing instead of a foreign body response. Fine-tuning these biomaterials to induce scar-free wound healing is a major undertaking, with potential applications in multiple diseases and conditions. This review focuses on the understanding of the immune response to biomaterials to suite the clinical need of promoting endogenous regeneration, with emphasis on muscle and skin tissues. A limitation of this review is that detailed discussion of biomaterials is provided only for applications in muscle and skin injuries. Despite this, many of the same considerations can be employed for biomaterial-based treatments of other tissues.

## Wound Healing and Biomaterials

The process of wound healing consists of three main stages: an inflammatory phase, regenerative phase, and a remodeling/repair phase (Gonzalez et al., 2016). The result of wound healing can be largely categorized in two different outcomes: full restoration of function or a chronic failure to remodel. A failure to remodel results in chronic fibrosis and scar tissue formation, which can also lead to chronic inflammation at the site of injury (Oishi and Manabe, 2018).

The process of wound healing is heavily dependent on immune signaling cues, which help in clearing of dead tissue, mobilization of local stem cells, and remodeling of extracellular matrix (Oishi and Manabe, 2018). Immune signaling can be modulated by biomaterials, that can also act to stabilize the injury site and aid in promoting repair.

### Inflammatory Phase

The inflammatory phase is characterized by angiogenesis, deposition of collagen, production of a scaffold composed of extracellular matrix (ECM) proteins, cell growth, and myofibroblast contraction to minimize the size of the wound (Atala et al., 2010). Immediately after the trauma, blood fills the site of injury to achieve hemostasis (Tonkin et al., 2015). Circulating, inactive enzymes are activated to trigger the complement cascade and clotting (Medzhitov, 2008). Local immune cells such as mast cells and tissue-resident macrophages secrete chemokines, cytokines, vasoactive amines, eicosanoids, and products of the clotting and complement cascade to recruit immune cells and activate circulating plasma proteins (Medzhitov, 2008).

Activated platelets release platelet-derived growth factor (PDGF), CXCL8, TNF-α, and other immune mediators (Ellis et al., 2018). This enables immune cell recruitment and further inflammation. Additionally, activated platelets adhere to ECM proteins at the injury site and aid in formation of a clot to prevent excess blood loss and pathogen entry (Nurden et al., 2008). The clot is also formed of ECM proteins such as fibronectin, vitronectin, and more, which act as attachment points for migrating cells entering the injury site (Barrientos et al., 2008).

In addition to platelet activation, inflammation is triggered by injured host cells *via* release of immune mediators and breakdown of ECM (Medzhitov, 2008). The best-established pro-inflammatory ECM component is glycosaminoglycan hyaluronate, which activates Toll-Like Receptors (TLRs), which activate inflammatory cascades. Release of Danger Associated Molecular Patterns (DAMPs) such as alarmins induce recruitment of immune cells, particularly macrophages, to the site of the wound (Bianchi, 2007). Any pathogens that invade the wound additionally release pathogen-associated molecular patterns (PAMPs) which are recognized by tissue resident cells and trigger further inflammatory signaling *via* TLRs (Ellis et al., 2018).

Mast cells are often found at the injury site, which activate to release cytoplasmic granules filled with inflammatory mediators such as histamine (Raziyeva et al., 2021). It is believed that mast cells help promote neutrophil infiltration at the injury site, and persistence of mast cells at the injury site is correlated with elevated levels of scarring at the injury site (Wulff and Wilgus, 2013). As a result, is believed that mast cells function at the wound site to augment the inflammatory phase of wound healing, and apoptose or leave the injury site when the process of wound healing progresses (Wulff and Wilgus, 2013).

Neutrophils are one of the first immune cell types to extravasate and respond to the inflammatory signaling at the site of the injury (Ellis et al., 2018). In particular, CXCL8 acts as a chemoattractant and recruits neutrophils to the site of injury. Neutrophils also are capable of producing CXCL8 to create a positive feedback loop (Ellis et al., 2018). Once at the site of injury, neutrophils aid against invading pathogens *via* release of cytoplasmic granules and reactive oxygen species. They also aid to promote angiogenesis *via* release of pro-angiogenic factors such as VEGF, aid in recruitment of other immune cells such as monocytes *via* release of MCP-1, TNF-a, and more, and tissue remodeling *via* release of uPA (Ellis et al., 2018).

Other innate immune cells, such as basophils, also contribute to the inflammatory phase of wound healing (Raziyeva et al., 2021). Basophils, recruited by chemokines such as GM-CSF, secrete large volumes of IL-4, a cytokine that promotes fibroblast and macrophage activation (Ellis et al., 2018; Raziyeva et al., 2021).

Monocytes/macrophages are a key cell type in the inflammatory phase of wound repair (Minutti et al., 2017). While tissue-resident macrophages are activated by local inflammatory cues post-injury, monocytes extravasate and enter the injury site *via* inflammatory cues and then polarize into different phenotypes (Hesketh et al., 2017). While the phenotypical classifications of macrophages is a debated topic, it is largely accepted that macrophages lie on a spectrum of polarization between M1 “proinflammatory” macrophages and M2 “anti-inflammatory” macrophages.

Macrophage polarization is largely determined by extracellular cues in the microenvironment. As monocytes enter the site of injury, the inflammatory cues they receive induce polarization into the M1 phenotype (Krzyszczyk et al., 2018). As inflammatory macrophages, they phagocytose dead tissue and secrete cytokines that promote inflammation and recruit more immune cells such as natural killer cells, macrophages, and T helper cells (Krzyszczyk et al., 2018). They additionally phagocytose apoptosed neutrophils to help clear the wound (Ellis et al., 2018).

If the innate part of the immune system is not sufficient in clearing the wound, the inflammatory phase persists with larger infiltrate of monocytes that differentiate into macrophages and dendritic cells (Medzhitov, 2008; Muire et al., 2020). Differentiation of monocytes into dendritic cells enables antigen presentation to adaptive immune cells and, particularly, activation of CD4^+^ T helper cells (Muire et al., 2020). In particular, CD4^+^ CD25^+^ T helper cells are found at the site of healing in various tissues (Campbell and Rudensky, 2020). However, there are also Th1, Th2, and Th17 helper cells found at the sites of tissue injury (Gause et al., 2013; Brockmann et al., 2017; Raziyeva et al., 2021). This will be elaborated on in the regenerative phase of wound healing.

### Regenerative Phase

The regenerative phase is characterized by closure of the wound, angiogenesis, and replacement of the inflammatory scaffold produced by coagulation by ECM proteins and fibroblasts (Gonzalez et al., 2016). Revascularization of the wounded site is a crucial part of regeneration, as the local tissue requires a source of nutrients. As blood vessels regenerate, a population of pericytes mediate their production and stability (Takakura, 2006). Interestingly, pericytes (also known as perivascular stem cells, or PVSCs) can also differentiate into cartilage, bone, or muscle precursors (Gonzalez et al., 2016). As a result, they play a crucial role in tissue repair. Cytokines and growth factors within the injury site recruit and activate fibroblasts, which secrete large volumes of ECM and contract further to completely seal the wound. The ECM components produced by fibroblasts, namely collagens, replace the hematoma formed in the inflammatory phase (Gonzalez et al., 2016). Precursor tissue cells began to rapidly proliferate and invade the margins of the wound site, to prepare for creation of fully functionalized tissue that will replace the ECM scaffold (Muire et al., 2020).

Macrophages play a crucial role in promoting endogenous repair of the site by directing stem cell activation and self-renewal (Chazaud et al., 2003; Minutti et al., 2017). In time, macrophages change from a proinflammatory phenotype to a regenerative, immunosuppressive phenotype (Oishi and Manabe, 2018). Not only does this aid in the transition from the inflammatory to the regenerative phase of tissue repair, but also provides cues for self-renewal and differentiation of the local stem cells (Arnold et al., 2007). By phagocytosing dead cell debris and promoting local stem cell function, macrophages play a crucial role in the transition from the regenerative to the repair phase of the healing process.

An important component of the regenerative phase is the tissue’s adult stem cell population, which works to replace the damaged site with fully functional tissue that is histologically identical to the site prior to injury (Mann et al., 2011). Stem cells are characterized by their ability to self renew and differentiate into different cell populations (Dekoninck and Blanpain, 2019). Every tissue has a different regenerative capacity-skin, for instance, is capable of faster regeneration than neuronal tissue (Iismaa et al., 2018). While regenerative capacity is tissue-dependent, stem cell mobilization and function is highly dependent on immune cell dynamics. For the purpose of this review, we will be focusing on how the immune system regulates stem cells and their function in wound healing.

As mentioned previously, adaptive immune cells begin to infiltrate the site of injury and also act in various ways to promote wound healing processes. CD4^+^ T cells, upon entry to the wound, polarize into different phenotypes depending on the cytokine signaling at the time of antigen presentation *via* antigen presenting cells such as macrophages and dendritic cells (Martinez-Sanchez et al., 2018). While there are multiple different subsets of CD4^+^ T cells, the role of Th2, Th17, and Tregs are well established as playing a role in wound healing.

Th2 helper T cells are characterized by their expression of transcription factor GATA3 and production of IL-4, IL-5, and IL-13 (Raphael et al., 2015). Research on Th2 helper cells in wound healing is particularly well established in the context of parasite-induced tissue damage, such as those that come as a result of helminth infections (Allen and Wynn, 2011; Gause et al., 2013). While M1 macrophages and other pro-inflammatory cascades are effective at clearing the injury site of any invading pathogens, they are not effective at inducing wound healing and resolution of inflammation (Allen and Wynn, 2011). As a result, it is theorized that Th2 helper cells evolved to provide this type of brake on inflammation, by secreting IL-4 and IL-13 to promote repair and polarize different cell types to a more immunosuppressive phenotype. This includes promoting fibroblast activation/secretion of ECM and M2 macrophage polarization (Allen and Wynn, 2011).

Th17 cells are characterized by their expression of transcription factor RORγt and production of IL-17 and IL-22 (Raphael et al., 2015). Their role in wound healing appears to be very dependent on the tissue and location of injury, largely due to the pleiotropic effects of IL-17. For instance, IL-17 has been shown to work synergistically with FGF2 to promote epithelial lining repair in a mouse model of colitis (Song et al., 2015). Nonetheless, the inflammatory properties of IL-17 have also been demonstrated to hinder wound healing, as IL-17 knockout mice experience better skin wound healing than wild-type mice (Rodero et al., 2013).

While the role of IL-17 in wound healing is a grey area, IL-22 production by TH17 cells is known to promote wound repair in many tissues by regulating fibroblast activation and production of ECM (Brockmann et al., 2017). IL-22 has been shown in mice to promote wound repair in the skin, intestine, liver, and kidney (Arshad et al., 2020).

Regulatory T cells are believed to act to oppose TH17 cells to suppress the residual inflammation (Raphael et al., 2015; Muire et al., 2020). Regulatory T cells can be found in circulation and also as tissue resident cells, both of which are known to play an important role in wound healing (Zaiss et al., 2019). Local release of epidermal growth factor-like growth factor Amphiregulin by tissue-resident Tregs actively aids in resolving inflammation *via* local release of Transforming Growth Factor Beta (TGF-B) (Zaiss et al., 2019). Hui et al. (2017) also demonstrated that, in zebrafish, Tregs infiltrated injured organs and secreted organ-specific regeneration factors such as Insulin-like growth factor in the retina. Furthermore, knockout of regulatory T cells severely impaired the regenerative capacity of the heart, spinal cord, and retina of zebrafish, independent of the immunomodulatory capacities. Secretion of immunosuppressive cytokines by regulatory T cells has known to also promote tissue healing after heart attacks and protect against kidney ischemia-reperfusion injury in mouse models (Kinsey et al., 2009; Weirather et al., 2014). Additionally, regulatory T cells actively promote local cell differentiation and restoration of homeostasis in certain contexts. In a mouse model of demyelination, regulatory T cells were shown to actively promote myelin regeneration and oligodendrocyte differentiation (Dombrowski et al., 2017). The combination of ECM deposition, transition to an anti-inflammatory and pro-regenerative immune state, and promotion of local stem cell activity are crucial components of the regenerative phase.

### Repair Phase

The repair phase of wound healing involves formation of functional tissue that is physiologically identical to the site prior to injury. Stem cells continue to differentiate and utilize the deposited ECM to direct the proper tissue formation. Extracellular matrix proteins such as fibronectin provide attachments for cells to bind to and promotes migration into the deposited ECM (Yamada, 2000). In time, the blood vessels, fibroblasts, and inflammatory cells in the area either exit the tissue or undergo cell death *via* apoptosis/or by other unestablished mechanisms (Gonzalez et al., 2016).

Regeneration or repair are two outcomes of a wound (Oishi and Manabe, 2018). Regeneration results in full resolution of the injury, with the resulting tissue being conformationally identical to the pre-trauma tissue. When a tissue is unable to fully regenerate, the body responds to a chronic fibrotic stage in which a scar forms and the regenerative phase does not cease (Atala et al., 2010). The repair outcome of a wound leads to development of a scar that can severely affect tissue function and leave patients with permanent and costly conditions. The cost to treat a diabetic foot ulcer episode (DFU) in the clinic was found to average $24,226 (Hicks et al., 2019). In 2014, an estimated 14.5% of medicare beneficiaries had at least one wound/wound-related infection, with Medicare spending on treatment of these wounds costing a minimum of $28.1 billion dollars (Nussbaum et al., 2018). With the aging population of the United States, this number is believed to increase over time unless better therapeutics are developed (Nussbaum et al., 2018).

### Use of Biomaterials in Wound Healing

Scientists have focused on developing ways to promote regeneration over repair, including creation of biomaterial scaffolds to support and guide this process. The ideal scaffold should meet the mechanical and physical needs of the native tissue, degrade as the new tissue is formed, integrate with the host tissue, and be reliably produced on a large scale (Sheikh et al., 2015). However, recent studies have also emphasized the need for the biomaterial to stimulate the regenerative capabilities of the immune system (Andorko and Jewell, 2017).

The immune system provides signaling cues for local stem cells and plays a crucial role in clearing the injury site (Strbo et al., 2014). This signaling is reviewed extensively elsewhere, but includes pro-regenerative signals such as Insulin-like Growth Factor-1 or pro-repair signals like TGF-B (Liu et al., 2016; Alcazar et al., 2020). The immune system is also incredibly sensitive to exogenous cues and can be utilized therapeutically to produce certain outcomes. By utilizing the immune system *via* biomaterials, the process of wound healing can be optimized, and the burden of long-term physical trauma can be ameliorated.

## Foreign Body Response

**FIGURE 1:**
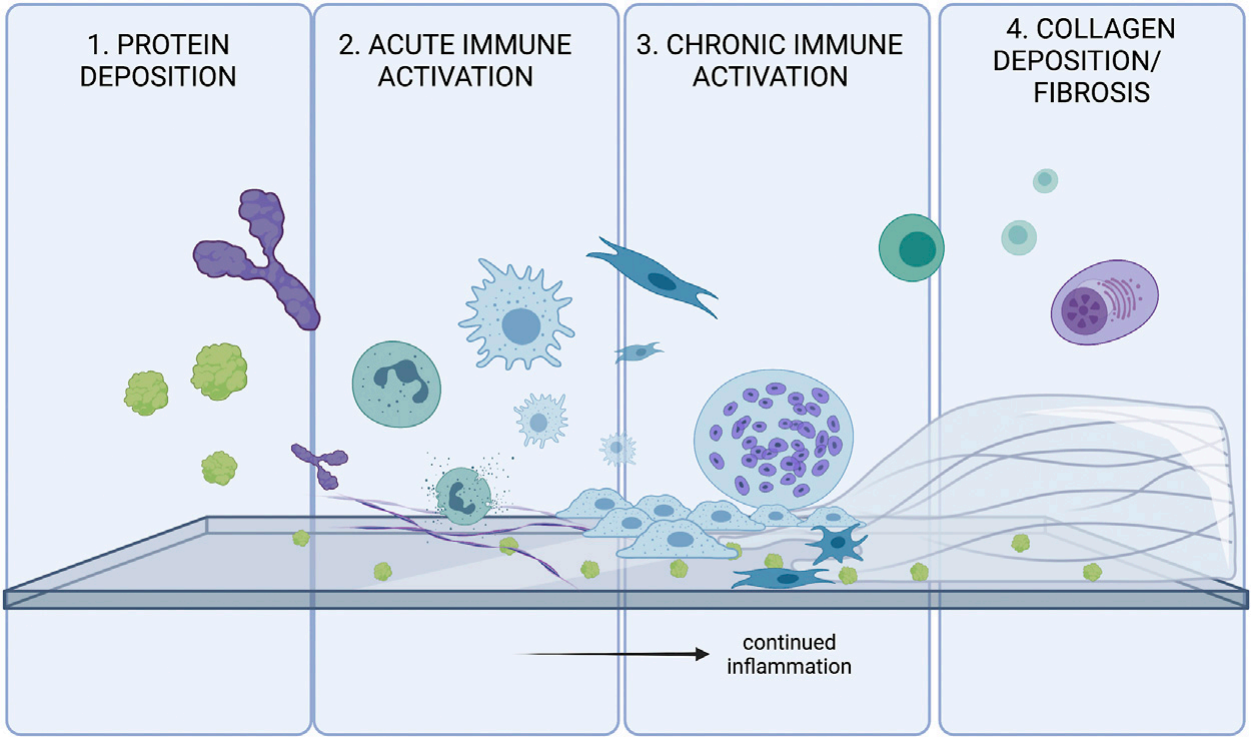
The four stages of the Foreign Body Response (FBR). FBR is initially triggered by protein adsorption. and activation of various inflammatory cascades. Innate immune systems attempt to degrade/phagocytose the foreign body, and will continue to try eliminating the threat until collagen accumulates on the surface of the foreign body. The fibrosis acts as a barrier to protect the body from the threat of the foreign body.

While biomaterials have great clinical promise, they can also have some off-target effects. Regardless of the classification of the biomaterial, virtually all biomaterials are capable of eliciting a foreign body response (Aramwit, 2016). There are four main steps of the Foreign Body Response: protein adsorption, acute inflammation, chronic inflammation, and collagen deposition around the implanted material (Zhang et al., 2021). These steps are outlined in Figure 1.

### Protein Adsorption

Upon implantation, proteins in the interstitial fluid and blood immediately bind to the material, triggering downstream immune pathways. These proteins include but are not limited to complement proteins, clotting proteins, or immunoglobulins (Williams, 2008). There is no one protein that is deemed solely responsible for a foreign body response. However, there are some that are found more commonly on the surface of a biomaterial. Albumin, fibrinogen, fibronectin, C3 protein, and gammaglobulins are all implicated in the foreign body response (Anderson et al., 2008). These proteins can undergo conformational changes and trigger proinflammatory signaling cascades such as the clotting and complement cascades (Mödinger et al., 2018). These cascades induce local inflammation and recruit mast cells, neutrophils, and macrophages to the biomaterial surface.

### Acute Inflammation

Neutrophils are one of the first cells recruited to the biomaterial site, as they are often the first cell to respond to inflammatory signals (Anderson et al., 2008). Mast cells are also a commonly found cell at the biomaterial surface. Both these cells degranulate to release more pro-inflammatory signals. IL-4 and IL-13 production by mast cells are known to play important roles in determining the extent of foreign body response mounted against the biomaterial (Anderson et al., 2008). Of all cell types, macrophages are one of the most crucial components of the Foreign Body Response (Williams, 2008; Sheikh et al., 2015; Mariani et al., 2019). Macrophages can bind to fibrinogen, fibronectin, and vitronectin *via* integrin receptors and thus latch on to the surface of the biomaterial (Anderson et al., 2008). IL-4 and IL-13 produced by mast cells drive macrophage activation (Sheikh et al., 2015).

### Chronic Inflammation

Upon binding to the biomaterial surfaces, macrophages immediately attempt to phagocytose the biomaterial (Medzhitov, 2008). When the size or shape of the biomaterial makes it impossible to do so, macrophages become frustrated and form Foreign Body Giant cells (FBGCs) to attempt to further phagocytose the site. IL-4 and IL-13, two cytokines often present at the biomaterial interface, are known drivers of Foreign Body Giant Cell formation (McNally and Anderson, 1995; DeFife et al., 1997).

FBGCs are terminally differentiated and not as plastic as macrophages, and contain high numbers of lysosomes (Sheikh et al., 2015). They are known to attach to the biomaterial surface and release a host of inflammatory cytokines and Reactive Oxygen Species (ROS) (Hernandez-Pando et al., 2000; Anderson et al., 2008). The cytokines expressed and the formation of FBGCs are largely determined by the biomaterial’s properties such as hydrophobicity or rigidity (Carnicer-Lombarte et al., 2021).

In addition to innate immune cells, there has been evidence that biomaterial-specific, adaptive immune cells are primed by Antigen Presenting Cells in secondary lymphoid organs (Van Luyn et al., 1998; Chung et al., 2020; Adusei et al., 2021). Various classes of T cells contribute to the Foreign Body Response, including TH1 and TH2 type T helper cells. Both cell types act largely by stimulating macrophages by releasing cytokines, Interferon Gamma (IFNy) by TH1 cells and IL-4 and IL-13 by TH2 cells (Adusei et al., 2021). Th17 cells and production of IL-17 by nonconventional gamma-delta T cells has also been implicated in driving of the foreign body response (Wolfram et al., 2012; Chung et al., 2020).

### Fibrous Capsule Formation

Upon unsuccessful phagocytosis of the biomaterial, the body forms a fibrous capsule around the biomaterial to block it off from the body (Anderson et al., 2008). Both M1 and M2 macrophages are believed to play a role in activating fibroblasts and promoting ECM secretion around the site (Witherel et al., 2019). Particularly, cytokines including VEGF, IL-6, TNF-alpha, and TGF-B1 are believed to be released by macrophages and promote fibrosis around the implanted biomaterial.

The fibrotic capsule around the implanted biomaterial is designed to protect the body from any harm the biomaterial may cause. Fibroblasts are recruited to the site by local inflammatory mediators, and their activation into myofibroblasts is characterized by production of alpha smooth muscle actin (a-SMA) (Witherel et al., 2019). The activated cells then produce large amounts of ECM on the surface of the material to block it off from the body. Monocyte coculture with various medical polymers and fibroblasts demonstrated that human IL-1 was responsible for monocyte-mediated fibroblast stimulatory potential (Miller and Anderson, 1989). Furthermore, the fibrous capsule around the biomaterial also consists of FBGCs, which continue to try and eliminate the biomaterial even after it is blocked off from the body (Anderson et al., 2008).

### Mitigating the Foreign Body Response

It is estimated that the conservative failure rate of implants due to foreign body response is around 10%, and the cost of solving this need is around $10 billion per year (Zhang et al., 2021). There are countless approaches to mitigating the foreign body response, including use of endogenously found substances for biomaterials, changing the physical properties of the biomaterial, and codelivery of anti-inflammatory drugs.

Tissue-derived ECM scaffolds have long been used due to its natural role in promoting endogenous repair and proving to be well suited for the clinic (Hortensius and Harley, 2016). Despite the inherent biocompatibility of ECM proteins, however, studies indicate that collagen implants can elicit a foreign body response (Aamodt and Grainger, 2016). This shows that ECM matrix and derivatives could adversely affect the healing processes in some unknown ways. However, the collagen often degrades before any adverse effects due to the foreign body response are really seen.

Plant-derived alginates (a polysaccharide block co-polymer of β-D-mannuronate and α-L-guluronate) also elicit a severe foreign body response (Doloff et al., 2017). Since it is not degradable like collagen, a fibrous layer will coat the biomaterial and prevent proper function. Veiseh et al. (2015) demonstrated that the foreign body response to spheres made of SLG20, an alginate where over 60% of the monomer units are guluronate, is inversely correlated with the diameter of the spheres. Interestingly, this seems to be the case for a variety of materials, including steel, glass, polycaprolactone, and polystyrene (Veiseh et al., 2015).

In addition to changing the shape and size of the biomaterial, alginates are extremely easy to chemically modify. Papers such as that of Vegas et al. (2016) demonstrate that alginates can be chemically modified to evade the immune response. Doloff et al. (2017) highlighted the importance of CSF-1 in recruitment of immune cells to the implanted biomaterial and demonstrated that blocking the CSF-1 receptor prevented the foreign body response in non-human primates. CSF-1 receptor is a key regulatory of myeloid cell activation, and its blockage essentially mitigates the myeloid response to the biomaterial (Stanley and Chitu, 2014). Regulating the myeloid response to biomaterials, therefore, is a promising approach to mitigating foreign body response.

Regardless of chemical composition, however, the size and morphology of the biomaterial itself could lead to a foreign body response. Padmore et al. (2017) showed a linear correlation between length of glass fibers with production of Interleukin 1-a, COX-2, and TNF-a by alveolar macrophages. Additionally, studies analyzing the effect of biomimetic multi-scale wrinkles demonstrate that wrinkled biomaterials promoted an anti-inflammatory macrophage phenotype and minimized the extent of collagen deposition in comparison to flat materials (Wang et al., 2016) Therefore, it is incredibly important to consider the multitude of variables that determine the extent of the foreign body response to a specific biomaterial.

## Muscle

Muscle tissue is highly structured and composed largely of long, multinucleated muscle fibers that are enclosed into bundles by extracellular matrix protein. Muscle precursor cells, named satellite cells, the resulting tissue is strong but dynamic, and able to meet evolving physical demands.

According to the World Health Organization, there are 1.71 billion people in the world suffering from musculoskeletal disorders (Cieza et al., 2020). Prevalence of musculoskeletal disorders is rising, though a majority of these cases are treated with physical rehabilitation and minimal need for intensive care (Muire et al., 2020).

Courtesy of the regenerative nature of muscle tissue, healthy muscle is often able to heal in response to minor trauma. Significant issues come, however, when the extent of muscle trauma exceeds the regenerative capacity of the muscle. This triggers a dysregulated immune and regenerative response that involves scarring, deteriorated muscle function, and long-term health issues (Andrea Sass et al., 2018). A common example of this kind of injury is the volumetric muscle loss (VML), which involves damage to muscle adjacent to a fractured bone (Hurtgen et al., 2016). Often, VML injuries are left untreated due to the lack of viable therapies.

### Muscle Healing

**FIGURE 2:**
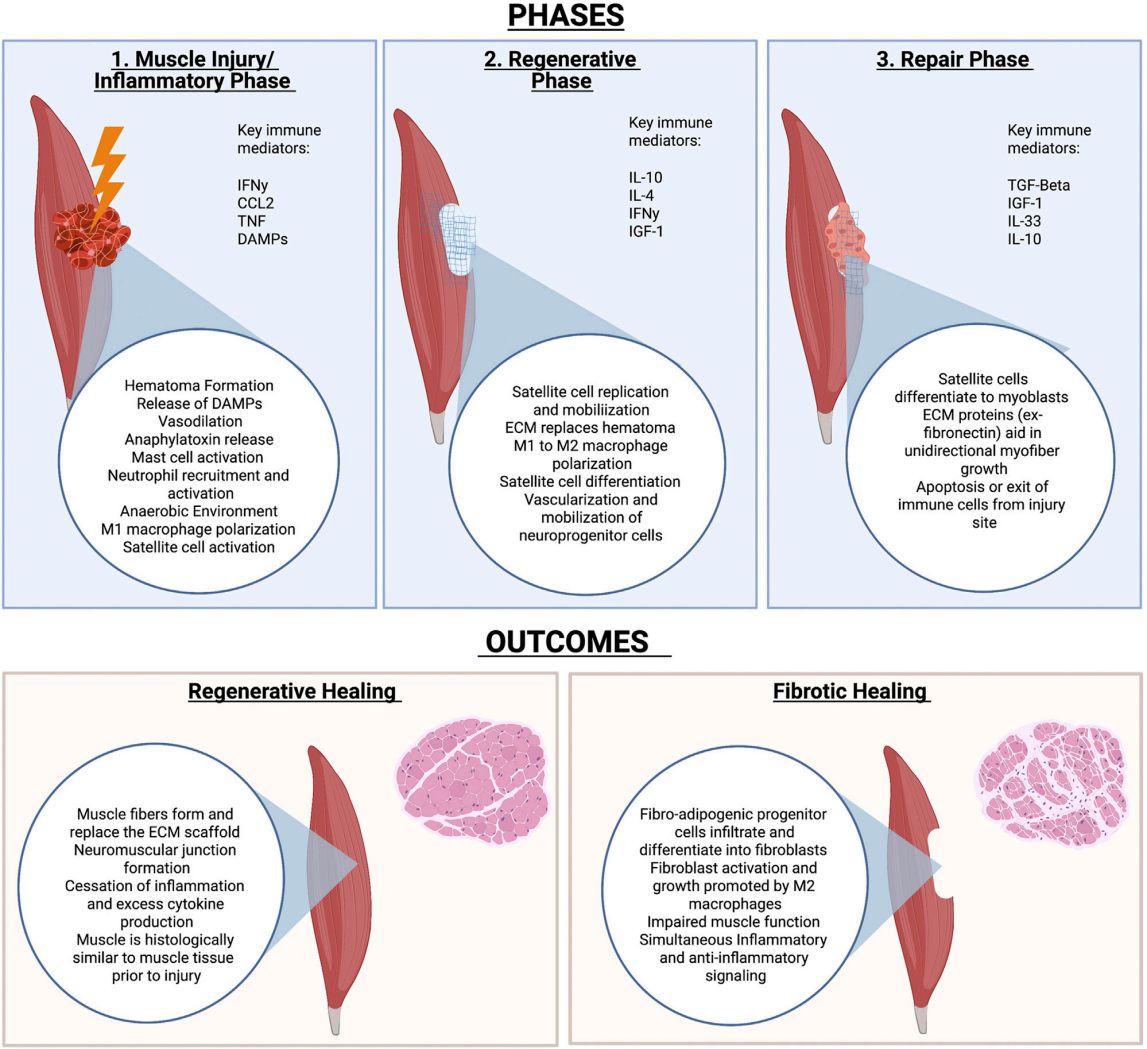
Phases of muscle healing and potential outcomes. Muscle Injury is largely characterized by an inflammatory, regenerative and repair phase. The outcome of injury depends on many factors but includes either full regeneration of injured tissue or fibrosis of the tissue that results in impaired function.

The process of muscle healing is extensively reviewed elsewhere (Muire et al., 2020) and briefly outlined in Figure 2. As mentioned previously, the healing process can be largely categorized into three major phases: the inflammatory, regenerative, and remodeling phases. The three phases are outlined in Figure 2.

The importance of the immune system in muscle homeostasis is emphasized by the estimated 10^9^ leukocytes per liter in adult, rodent limb muscles (Tidball, 2017). Upon muscle injury, the site of injury fills with blood and a scaffold-like hematoma forms to recruit immune cells and protect the tissue from infection (Muire et al., 2020).

Activated platelets, neutrophils, and more recruit monocytes that activate to become macrophages (Muire et al., 2020). M1 macrophages and other innate immune cells act to clear the site. Once the clot is formed and there is no threat of pathogens, the immune system begins to focus on repair of the tissue instead of inflammation. This includes polarization of immune cells such as macrophages and T cells to a more immunoregulatory phenotype. M2 macrophages and TH2 T helper cells, in particular, promote myofibroblast activation to allow for ECM production and replacement of the hematoma with ECM. The ECM deposition and modelling acts to stabilize the wound site and promote proper myofiber growth to conform to the original tissue. This ensures formation of a muscle that is morphologically identical to the uninjured tissue (Muire et al., 2020).

In addition to promoting ECM deposition at the injury site, macrophages play a key role in muscle stem cell function. Muscle stem cells are present as satellite cells, which rapidly self-renew and then fuse together to form myofibers (Muire et al., 2020). While satellite cells are the main myofiber precursors, it is also important to note that perivascular stem cells (PVSC) located adjacent to blood vessels in skeletal muscle can also differentiate into myoblasts (Sicari et al., 2014a). It is well established that M1 macrophages promote satellite cell replication, while M2 macrophages induce M2 differentiation (Dziki et al., 2016). Furthermore, direct coculture of macrophages with muscle precursor cells decrease apoptosis of the precursor cells (Chazaud et al., 2003).

As myoblasts form into myofibers, the cells rely on ECM to guide their differentiation and rely on mechanical cues from ECM during this process (Muire et al., 2020). Interestingly, mechanical stimulation appears to promote the crosstalk between M2 macrophages and myoblasts during muscle formation in mice (Dziki et al., 2018).

When the injury is too large, ECM secretion persists as immune cells fail to properly regulate ECM deposition and myoblast differentiation. Excess deposition leads to failure of satellite cells to form proper myofibers, resulting in scar tissue formation instead of muscle tissue. Adipose tissue and fibroblasts invade the injured space, and seal the wound to protect the body from invading pathogens (Muire et al., 2020).

Traumas like VML injuries are immediately predisposed to a failure of resolution due to the massive immune cell influx that comes as a result of release of immune mediators by the injured tissue (Bianchi, 2007). Namely, Damage Associated Molecular Patterns (DAMPs) and Alarmins are highly elevated following a traumatic muscle injury. Satellite cells require mechanical stimulation to differentiate into muscle cells, so efforts to create biomaterials for muscle injuries have largely relied on creating scaffolds that aid in mechanical support for satellite cells. Additionally, a large focus of these materials is promotion of immunoregulation to prevent the continuation of inflammation that ultimately leads to fibrosis. These types of materials can be broadly characterized into Extracellular Matrix Proteins and derivatives, natural biomaterials, synthetic biomaterials, and cell-laden biomaterials. Common biomaterials, their immunomodulatory properties, and potential clinical obstacles are outlined in Table 1.

**TABLE 1:** Biomaterials for skeletal muscle tissue regeneration and their immunomodulatory properties.

Class	Name	Type of muscle injury	Immune modulation	Publication
ECM SCAFFOLDS/DERIVATIVES	Xenogeneic ECM Scaffolds	Volumetric Muscle Loss	Promotes M1 to M2 macrophage transition	Hoganson et al. (2010); Sicari et al. (2014a); Sadtler et al. (2016)
			M2 macrophages promote PVSC and neurogenic precursor cells migration	
			ECM induces IL-4 production to promote Th2 T helper cell polarization (essential for wound healing)	
			Induces release of VEGF, and IGF-1 to induce myogenesis and regulate inflammation	
	Hyaluronic acid	Primary mouse cells derived from tibialis anterior muscle	High molecular weight HA is known to promote cellular invasion and differentiation of epicardial cells *via* CD44 and MEKK1	(Craig et al., 2010; Jha et al., 2015; Silva Garcia et al., 2019; Schuurmans et al., 2021)
			High MW heparin retains growth factors and releases them slowly to promote regeneration and immune modulation (ex- TGF-B)	
			Can be modified to include ECM components (ex- laminin and RGD peptides) promote invasion of myoblasts into hydrogel	
	Minced Muscle Grafts	Volumetric Muscle Loss	Significantly reduced cell infiltrate into injury site	Hurtgen et al. (2017)
			Graft elevated production of MCP-1, IL-10, and IGF-1	
			Minimized circulating levels of RAGE and other alarmins	
Naturally-Derived Polymers	Alginate	Volumetric Muscle Loss	Alginate ferrogel planted near the site of injury was used to induce mechanical compression; mechanical stimulation of cells + clearance of inflammatory mediators promoted muscle healing	Cezar et al. (2016); Sun and Tan (2013); Doloff et al. (2017)
			Can be chemically modified to inhibit immune reactions or promote specific cell activity (ex- CSF-1 inhibitors, RGD)	
	Silk Fibroin	*In-vitro* (human skeletal muscle myoblasts)	Silk fibroin scaffolds can promote myoblast ECM deposition and promote myofiber formation	Thurber et al. (2015); Chaturvedi et al. (2017)
Synthetic Biomaterials	Polyprolene mesh	Abdominal Wall Defect/Hernia	Has the mechanical properties desired for many medical applications; can be coated with ECM to prevent foreign body response and promote repair *via* M2 macrophage polarization	Wolf et al. (2014); Wang See et al. (2020)
	Polyethylene Glycol (PEG)	*In-vitro* (Rat and Human Aortic Muscle Cell Lines)	Can be engineered to be proteolytically degradable and avoid foreign body response	Mann et al. (2001); Han et al. (2019)
		Cryo-injured tibialis anterior injury	Can be loaded with growth factors to induce immune effects and regeneration	
	Methacrylic Acid (MAA)	Intramuscular Injection	Promotes M2 macrophage polarization *via* IGF-1	Lisovsky and Sefton (2016); Talior-Volodarsky et al. (2017); Carleton and Sefton (2019)
			Promotes sonic hedgehog signaling to increase vascularization and wound healing	
			Upregulation of Arg and Fizz1	

### Extracellular Matrix Proteins/Derivatives

Extracellular matrix (ECM) and derivatives are a well-established class of biomaterials and have already shown great clinical success (Aamodt and Grainger, 2016). At its purest form, ECM biomaterials are protein-based, decellularized extracts from animal tissue and are largely composed of Collagen I by weight.

Extracellular matrix (ECM) and derivatives have already shown great clinical success in treating muscular injury (Aamodt and Grainger, 2016). Cheap, easy to produce, and effective, ECM biomaterials are protein-based, decellularized extracts from animal tissue. Type I Collagen is the main component of decellularized ECM, and has been used successfully for decades since it is readily degraded by the body (Aamodt and Grainger, 2016). This ensures that whatever foreign body response to the collagen is minimal.

While Type I collagen is not immunogenic, it is the various smaller components of ECM that succeed in triggering desirable cell stimulation, activation, or migration. This includes growth factors, ECM degradation products, or cell adhesion molecules (Dziki et al., 2017).

Porcine ECM scaffolds have been shown to promote muscle wound healing in mice and humans (Sicari et al., 2014b). Deeper research into the mechanism of action demonstrate that immune modulation by ECM scaffolds aids in the promotion of muscle repair.

Hoganson et al. (2010) demonstrated that cytokines encapsulated in porcine mesothelium aid in muscle repair, namely Vasoendothelial Growth Factor (VEGF), Insulin-like growth factor (IGF-1), and Transforming Growth Factor Beta TGF-β assay. This study also demonstrated that there was an unidentified factor released by porcine mesothelium that promoted VEGF production in fibroblasts, which is key in promoting revascularization of the wound.

Release of IGF-1 by ECM scaffolds also aids in M1 to M2 macrophage polarization during the transition from the inflammatory to repair phase in muscle wound healing (Tonkin et al., 2015). As mentioned previously, severe muscular trauma is likely to result in scarring as a result of the large amounts of alarmins released as a result of injury. Promoting transition of the inflammatory phase to the repair phase is a goal of many biomaterials designed to treat muscular trauma. Indeed, decellularized skeletal muscle ECM with encapsulated IGF-1 demonstrated increased muscle regeneration and minimized fibrosis in a rabbit animal model of VML injury (Lee et al., 2020).

In addition to M2 macrophages, myoblasts and neurogenic precursor cells were found to infiltrate to the center of the implanted ECM. Researchers previously demonstrated that degradation products from ECM scaffolds promote M2 macrophage phenotype similarly to IL-4 (Sicari et al., 2014a). M2 macrophages then promote chemotaxis and myogenesis of satellite cells and PVSCs into the center of the scaffold.

Sadtler et al. (2016) highlighted the importance of T cells in ECM-mediated endogenous repair of muscle by demonstrating the inability of RAG1 deficient mice to heal in the same capacity as wild type mice in a model of VML injury. In this experiment, mice underwent a volumetric loss injury in the quadriceps muscle, which was then filled with tissue-derived ECM due to their immunomodulatory properties. In RAG1 deficient and RICTOR deficient mice, the immunomodulatory properties of ECM scaffolds were not apparent. When comparing the immune cell phenotypes, immunomodulatory, regenerative macrophages were downregulated in the RAG1 mouse. Interestingly, levels of CD206+ macrophages were restored upon wild-type CD4^+^ transfer, but not with transfer of Rictr- T cells (Sadtler et al., 2016). Rictor is a component of the mTORC2 complex, which drives polarization of CD4 T cells to a Th2 phenotype. Sadtler et al. (2016) proposes that IL4 production initially induced by macrophages and innate immune cells is thus propagated by Th2 helper cells in the weeks after muscle injury, which drives healing and a pro-regenerative environment.

ECM scaffolds used for regenerative medicine have demonstrated varying results *in vivo* and highlight several important aspects of endogenous repair in muscle trauma. Human trials of porcine ECM transplanted into VML patients showed variable results (Sicari et al., 2014b). While 3 patients out of five experienced an increase in functional outcome variables, two saw no difference. Interestingly, the two patients with no improvement had the same injury type as a patient who improved, highlighting the variability of the outcomes. There are, however, several limitations to this trial. All patients enrolled in the trial had already gone through several rounds of physical therapy and were treated with ECM a year after injury. In contrast, the murine model in the paper received ECM treatment immediately after VML injury. It is unclear whether ECM treatment directly after injury could aid in VML repair.

Specific ECM components can be chemically modified to have different biological functions. Bencherif et al. (2008) created a photopolymerizable polymer made of hyaluronic acid and glycidyl methacrylate modified to include the RGD peptide of integrin, which promoted myoblast cell adhesion and differentiation *in vitro*. Expanding on this, researchers developed hyaluronic acid hydrogels chemically conjugated with peptide components of ECM, and showed that the CSGIKVAV peptide of laminin promoted cell adhesion and satellite cell migration into the scaffold (Silva Garcia et al., 2019).

In order to minimize the need for myoblast migration into the scaffold, researchers have demonstrated the efficacy of autologous minced muscle grafts in repairing VML injury in rats (Hurtgen et al., 2017). The addition of minced muscle grafts aided not only in restoring muscle function after injury to the Tibialis Anterior muscle, but also restored aggregation of mineralization at the fracture site of a neighboring bone. While promising, clinical feasibility of this therapy will rely heavily on finding an alternative source of muscle cells.

### Natural Biomaterials

As mentioned before, ECM biomaterials have limitations that might be solved by differently sourced biomaterials. Several naturally-derived polymers have shown great promise as biomaterials to promote regeneration. Materials such as agarose, alginate, silk, fibrin, and more have all been manipulated to treat VML.

Fibrin is a key component of the clotting cascade and is a natural part of the wound healing process (Grasman et al., 2015; Muire et al., 2020). Additionally, the fibrillary structure of Fibrin promotes myoblast survival, proliferation, and differentiation (Matthias et al., 2018). Muscle derived stem cells encapsulated in fibrin gels effectively differentiated into myofibers and promoted effective VML repair (Matthias et al., 2018).

Grasman et al. (2015) developed a scaffold consisting of biopolymer microfibers using fibrinogen and thrombin crosslinked by carbodiimide. They also adsorbed Hepatocyte Growth Factor (HGF) to the surface of fibrin microfibers and demonstrated that myofiber growth was increased with the implants. It is important to note, however, that collagen deposition remained the same across all groups, meaning that the extent of fibrosis may not have necessarily been decreased by the implant. This could likely be caused by a foreign body response or recruitment of fibroblasts by the adsorbed Hepatocyte Growth Factor (HGF).

Studies on the immunomodulatory effects of fibrin as a biomaterial differ-while some studies suggest that fibrin minimizes monocyte/macrophage recruitment, others demonstrate that fibrin gels increase leukocyte recruitment and promote production of proinflammatory cytokines IL-1B and IL-6 (García-García and Martin, 2019). Encapsulation of different cytokines within fibrin gels could help increase the immunomodulatory effects of fibrin gels and counteract undesirable immune stimulation by the biomaterial.

Alginate is another very popular biomaterial, due to ease of chemical modification, crosslinking, and biocompatibility. Alginate is derived from seaweed and is a polymer formed from (1–4)-linked β-D-mannuronic acid (M) and α-L-guluronic acid (G) monomers (Sun and Tan, 2013). Alginate is easily crosslinked to form a gel from a fluid using cations such as calcium and barium, meaning that there are minimal adverse effects to cells encapsulated within the alginate in comparison to other crosslinking methods such as ultraviolet photopolymerization. It is also very easy to chemically modify with various peptides to stimulate certain cell behavior, the most common of which being the RGD peptide from integrins (Sun and Tan, 2013).

Unlike other biomaterials for muscle healing, alginate is unique because it does not degrade easily in the body (Sahoo and Biswal, 2021). However, it has still been used in various wound healing contexts. Cezar et al. (2016) developed a ferrogel using an alginate-based scaffold containing 7% iron oxide. Instead of implanting directly at the injury site, the device was implanted subcutaneously and used a magnet to trigger mechanical compressions to stimulate the wounded muscle, as opposed to having a scaffold directly transplanted into the defect. A significant improvement in muscle healing and function with this was observed, likely due to mechanical stimulation of cells and physical clearance of inflammatory mediators like Reactive Oxygen Species (ROS) (Cezar et al., 2016). This work demonstrates that physical stimulation may aid in promoting the inflammatory to regenerative phase of trauma healing.

Silk biomaterials are an emerging trend in wound healing and regenerative medicine (Pollini and Paladini, 2020). Similar to alginates, silk biomaterials such as silk fibroin can be highly tunable and have shown great efficacy in promoting wound healing in the eye, nervous system, and more (Pollini and Paladini, 2020). Only recently have silk scaffolds been developed for muscle healing in particular. Chaturvedi et al. (2017) demonstrated that myoblasts can grow and differentiate effectively on various silk fibroin scaffolds. Interestingly, Chaturvedi observed that the silk aided in promoting deposition of ECM by the myoblasts and proposed that the method by which the fibroins were presented to the myoblasts mattered more than the chemical composition of the fibroins. There are few papers reporting *in vivo* function of silk biomaterials for muscle healing. However, reports indicate that there are certain derivatives of silk that are able to not trigger an adverse immune response and are able to be degraded by macrophages *in vivo* (Thurber et al., 2015).

### Synthetic Biomaterials

Because of the limitations of ECM-derived scaffolds, alternative biomaterials with similar morphological or physical characteristics are desirable. An added benefit to these materials is that many of them can be fine-tuned with a lot more accuracy and precision, making it easier to alter their mechanical and biological properties.

A key aspect of synthetic biomaterials is their ability to be formed into various configurations. Synthetic materials can be formed into meshes, foams, hydrogels, and electrospun scaffolds (Wolf et al., 2015). These configurations can determine the cellular response to the biomaterial, such as providing better attachment points for myoblasts (McKeon-Fischer et al., 2011). These kinds of materials include Poly (lactic acid) (PLA), poly (glycolic acid), Poly (e-caprolactone) (PCL), and more (Wolf et al., 2015).

Polyprolene mesh has been used for hernia repair for over 50 years and is considered the standard of care to reinforce the abdominal wall muscles after a hernia. Nonetheless, there are a host of issues with polyprolene mesh, resulting in complications reviewed by Gavlin et al. (2020). Two common complications of synthetic meshes are infection and fibrosis, triggering chronic inflammation and discomfort to the patient (Wang See et al., 2020).

Because myoblasts have mechanical requirements for adhesion and myotube formation, the rigidity that synthetic biomaterial makes them an ideal material for muscle regeneration. The biggest flaw of most synthetic biomaterials, however, is the inherent lack of immunocompatibility, and thus highlights the importance of this trait in a regenerative biomaterial. For instance, myoblasts seeded in sheets of Poly (glycolic acid) fiber mesh successfully formed unidirectional myofibers *in vivo* (Saxena et al., 1999). However, invading fibroblasts and foreign body giant cells overwhelmed the construct and makes the PGA fiber mesh alone unsuitable for transplantation.

Polyethylene glycol (PEG) is a common synthetic biomaterial, largely because it was believed to not be as immunogenic as other synthetic biomaterials and has many physical characteristics that make it a desirable biomaterial (Gombotz et al., 1991). PEG hydrogels containing a preoteolytically degradable peptide sequence and the RGD peptide promoted muscle cell adherence and migration into the hydrogel (Mann et al., 2001). Similarly, a PEG hydrogel loaded with Wnt7a, a promyogenic factor, promoted skeletal muscle migration and also promoted myotube formation from transplanted myoblasts in mice (Han et al., 2019).

Methacrylic Acid (MAA) has shown great efficacy as a biomaterial in other tissues, and has recently been applied to skeletal muscle. Methacrylic acid promotes IGF-1 signaling and increases presence of Fizz-1+ macrophages in skeletal muscle (Carleton and Sefton, 2019). MAA also promotes sonic hedgehog (SHH) signaling and promotes vascularization (Talior-Volodarsky et al., 2017).

Applying electrospinning techniques to synthetic biomaterials has significantly increased the feasibility of synthetic scaffolds for tissue engineering by making them less immunogenic. This technique allows for easy and tunable manipulation of synthetic biomaterials to create scaffolds with similar properties as the natural extracellular matrix (Sill and von Recum, 2008). Electrospun scaffolds can also incorporate various drugs such as growth factors, RNA, antibiotics, and more in a safe and reliable manner. The process of electrospinning is summarized in various papers, but results in formation of highly tunable microfibers of polymers (Sill and von Recum, 2008). By changing the voltage, distance between the needle and collector, viscosity, and feed rate can be optimized to change properties of the biomaterial (Politi et al., 2020).

Electrospinning has greatly improved efficacy of synthetic biomaterials for muscle healing. PLGA fiber scaffolds laden with myoblasts showed aligned myofiber growth and development, indicating that PLGA was a suitable biomaterial to promote muscle formation (Narayanan et al., 2020). PLGA is a generally well tolerated synthetic material that degrades to lactic acid and glycolic acid (Wolf et al., 2015). Yang et al. (2014) demonstrated that patches made of nanopatterned PLGA and myoblasts successfully formed myofibers in a model of muscular dystrophy. In the future, incorporation of immunomodulatory components to synthetic biomaterials could bolster the efficacy of these materials even more.

### Combinatorial Materials

As previously mentioned, each biomaterial type has its limitations. However, significant progress has been made in combining various materials to create a superior scaffold. While synthetic biomaterials may provide the best scaffolds for myoblasts to attach and differentiate upon, foreign body response to most of these materials is virtually unavoidable. While synthetic scaffolds have been used in the clinic for decades, permanent implantation of these materials alone to treat an area where fibrosis is likely to trigger adverse effects in many patients. As a result, combinatorial materials made of both immunomodulatory and synthetic scaffolds are an emerging field.

To combat the M1 macrophage-mediated response against polypropylene mesh, Wolf et al. (2014) coated the polyprolene mesh with an ECM hydrogel before implantation. The surrounding ECM was sufficient to combat the highly inflammatory nature of the polyprolene mesh, particularly by reducing the number of M1 macrophages and Foreign Body Giant Cells present around the mesh.

Using ECM scaffolds to combat the foreign body response to synthetic biomaterials has also shown success with PCL scaffolds. Jin et al. (2021) demonstrated the efficacy of a combinatorial biomaterial composed of PCL and muscle-derived ECM in promoting VML repair.

## Skin

### Skin Healing

Skin is comprised of two layers, the epidermis (upper layer) and dermis (lower layer), that are separated by the basement membrane. These layers are in turn composed of sublayers with different cell types and structures (Rognoni and Watt, 2018). The epidermis contains epithelial cells such as keratinocytes and Merkel cells, melanocytes, and immune cells such as Langerhans cells, γδT-cells, and CD8^+^ tissue-resident memory T cells, while the dermis contains dendritic cells, macrophages, innate lymphoid cells, natural killer (NK) cells CD8^+^ tissue-resident memory T cells, fibroblasts, and pericytes (Rognoni and Watt, 2018; Ho and Kupper, 2019). The immune and endothelial cell populations within the dermis have been shown to change with age (Rognoni and Watt, 2018).

**FIGURE 3:**
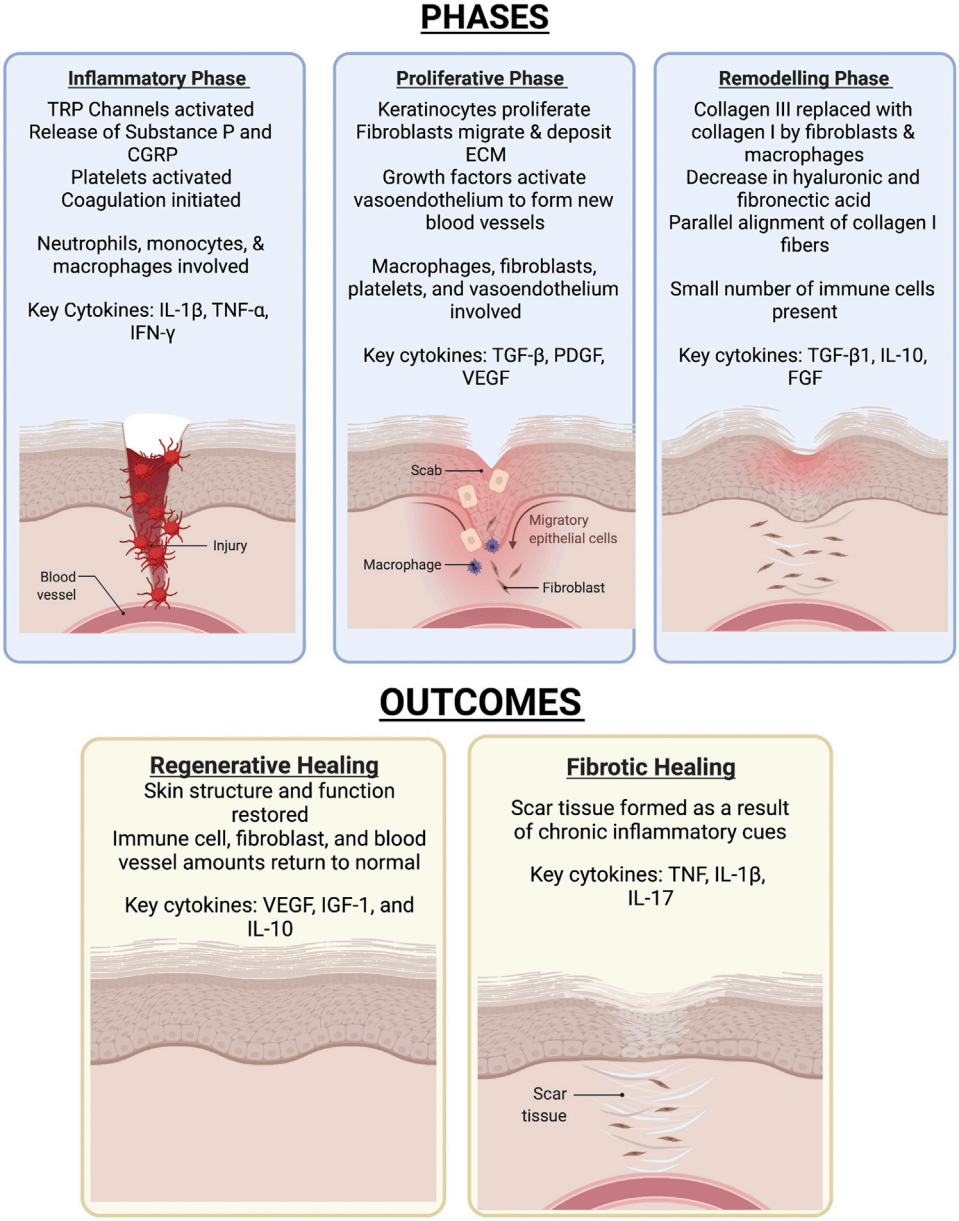
The various phases of skin wound healing and the outcomes. Skin healing consists of three phases-the inflammatory, proliferative, then remodelling phase. The outcome of the healing process results in either full resolution of injury or formation of a scar.

Detailed reviews of skin composition and the wound healing process have been written by others (Landén et al., 2016; Rognoni and Watt, 2018; Cañedo-Dorantes and Cañedo-Ayala, 2019). In brief, skin injuries trigger a three-phase process that has an inflammatory phase, proliferative phase, and a remodeling phase as seen in Figure 3. In the inflammatory phase, skin damage activates transient receptor potential (TRP) channels, stimulating sensory neurons to create action potentials (Gouin et al., 2017; Cañedo-Dorantes and Cañedo-Ayala, 2019). Many TRP channels such as those responding to temperature are expressed in skin keratinocyte cells (Patapoutian et al., 2009). This leads to the initiation of pain and the release of substance P and calcitonin gene-related peptide (CGRP) that play a variety of roles in increasing blood flow and vascular permeability, as well as attracting inflammatory cells (Schmelz and Petersen, 2001; Cañedo-Dorantes and Cañedo-Ayala, 2019). Indeed, the vasodilator activity of CGRP has been shown to be regulated by substance P (Brain and Williams, 1988). Vascular injury exposes blood platelets to basement membrane proteins such as collagen (Schmelz and Petersen, 2001; Bergmeier and Hynes, 2012), activating the platelets and leading to the initiation of the coagulation cascade. Platelet cell surface receptors interact with other cells and release growth factors to lead to pathogen detection and elimination (Tamagawa-Mineoka, 2015; Nami et al., 2016; Cañedo-Dorantes and Cañedo-Ayala, 2019).

The proliferative phase is characterized by fibroblast, keratinocyte, and endothelial cell migration and proliferation (Landén et al., 2016; Boothby et al., 2020). Keratinocytes respond to multiple signals and stimulatory factors by moving from the wound edge and proliferating to re-epithelialize the wound surface and rebuild the basement membrane (Jacinto et al., 2001; Barrientos et al., 2008; Landén et al., 2016). Fibroblasts from the surrounding area also migrate in response to multiple signals and stimulatory factors, leading to degradation of the provisional matrix and deposition of ECM components (Gill and Parks, 2008; Schultz and Wysocki, 2009; Nissinen and Kähäri, 2015; Xue and Jackson, 2015; Landén et al., 2016). Macrophage-fibroblast interactions in particular have been found to play an important role in myofibroblast proliferation and skin repair (Shook et al., 2018). This phase also involves angiogenesis to restore the tissue’s vascular network. Growth factors at the wound site activate endothelial cells from existing vessels, leading to their migration and formation of new blood vessels, which then recruit pericytes and smooth muscle cells (Li et al., 2003; Landén et al., 2016). Macrophages, fibroblasts, platelets, and endothelial cells each play important roles in the proliferative phase (Cañedo-Dorantes and Cañedo-Ayala, 2019). During the remodeling phase, deposited Collagen III is replaced by Collagen I (Landén et al., 2016).

Stem cells play an important role throughout the skin wound healing process, with involvement of both stem cell proliferation and signaling in healing (Coalson et al., 2019). Studies in mouse skin epidermis have revealed that stem cell activation during the wound healing process leads to the generation of new stem cells and progenitors that promote tissue expansion (Aragona et al., 2017). Adult stem cells including endothelial progenitor cells, bone marrow-derived mesenchymal cells, and adipose tissue-derived stem cells can each contribute to skin wound healing (Kim and Suh, 2010). Bone marrow-derived mesenchymal stem cells are known to differentiate into several types of skin cells including keratinocytes, endothelial cells, pericytes, and even monocytes/macrophages (Sasaki et al., 2008). Endothelial progenitor cells and adipose tissue-derived stem cells are each involved in revascularization of injured tissue (Kim and Suh, 2010). Extensive studies have been conducted to apply stem cells for skin wound healing (Nourian Dehkordi et al., 2019), but detailed discussion of approaches that do not make use of biomaterials are beyond the scope of the present review.

### Types of Skin Wounds and Biomaterial Approaches

The applications of skin wound healing include treatment of diabetic wounds, burn wounds, and full-thickness skin wounds. Traditional biomaterial-based approaches to skin wound healing typically focus on providing flexible mechanical properties while protecting the wound from infection. In this section, however, we will focus on biomaterial-based approaches that seek to modulate the body’s immune response to aid in the healing process. Many skin wound healing studies include the use of polymeric nanofibers [reviewed by Miguel et al. (2019)], the use of polymeric scaffolds [reviewed by Dickinson and Gerecht (2016), Sahana and Rekha (2018)], or the use of hydrogels. A number of immune-focused skin healing approaches aid in macrophage transition from a pro-inflammatory phenotype to anti-inflammatory phenotype, but some address multiple stages of the skin healing process as seen in Table 2. We categorize these approaches based on their use of naturally derived biomaterials or a combination of natural and synthetic materials. The use of synthetic materials alone is unlikely to be favorable due to an increased immune response to synthetic materials.

**TABLE 2:** Biomaterials for skin regeneration and their immunomodulatory properties.

Class	Name	Type of skin injury	Immune modulation	Publication
Combinatorial biomaterials	Sodium Alginate and Bioactive Glass hydrogels	Full-thickness skin wound	Macrophages promote migration of fibroblasts and mouse artery endothelial cells in skin	Zhu et al. (2020)
	Coaxial scaffolds of PLGA nanofibers + fibrinogen _ collagen I	Diabetic Wound	Combination of Fibrinogen + Collagen I exposure promoted M2 macrophage polarization and healed the wound by stopping inflammation	Sun et al. (2021)
	Stem cells loaded in biomaterials	Diabetic rat wound	Downregulation of pro-inflammatory cytokines; Increased M2/M1 ratio	Chen et al. (2021)
	Anti IL-6 eluting GelMA hydrogels	Mouse skin transplant	Promoted skin allograft survival by minimizing T cell and macrophage transplantation	Uehara et al. (2019)
	PEG hydrogels + Silver Ions and mangiferin liposomes	Mouse skin flap injury	Prevented infection, increased microvessel density, and the magniferin minimized macrophage recruitment	Mao et al. (2019)
	Microporous Annealed Particle Hydrogels with D-Amino acids	Mouse full-thickness incisional wounds	Increased immune cell recruitment lead to tissue regeneration and prevented scarring	Griffin et al. (2021)
			IL-33 production by local myeloid cells promoted healing	
Natural biomaterials	Keratin	N/A (*in vitro*)	Low molecular weight keratin promoted M1 to M2 transition in macrophages	Waters et al. (2018)
	Me-HA hydrogels loaded with bFGF	Mouse full-thickness skin wounds	Improved re-epithelialization, granulation formation, collagen deposition, skin appendage regeneration, and vascularization	Chen et al. (2020)
	Electrospun Soy Protein Scaffolds	Pig model of full thickness excisional wound	Dermal papillae formation in the dermis, collagen formation, and a well-formed stratified epithelial layer; Formation of dermal appendages	Har-el et al. (2014)
	Electrospun tilapia skin collagen membranes	Sprague Dawley rat full-thickness skin wound models	Improvement in wound healing rate and reduced inflammation compared to Kaltostat and untreated controls	Zhou et al. (2016)

### Naturally-Derived Biomaterials

Many skin healing studies have been undertaken with naturally-derived biomaterials (Hortensius and Harley, 2016), but few studies on skin healing have conducted extensive examination of the immunomodulatory functions of these naturally-derived biomaterials.

Collagen-based wound dressings are a naturally-derived biomaterial commonly used in treating skin wounds. Collagen, a highly-abundant extracellular matrix component, represents a family of glycoproteins that are involved in a variety of physiological functions. For example, collagen VII is known to lead to re-epithelialization through organization of laminin-332 at the dermal-epidermal junction, in addition to supporting dermal fibroblast migration and regulating cytokine production (Nyström et al., 2013). In addition to providing 3D structure and favorable mechanical properties, collagen interacts with cells *via* mediation by proteins that bind with the Pro-Hyp-Gly unit, those that bind with the Phe-Hyp-Gly sequence, receptors for collagen’s cryptic binding sites, and receptors for non-collagenous domains (Chattopadhyay and Raines, 2014). Zhou et al. (2016) derived collagen sponges from tilapia skin. Through *in vivo* implantation of the collagen sponges, they found that the material itself did not lead to immune activation. They further formed membranes by electrospinning the collagen sponges and determined that the *in vitro* adhesion and proliferation of human keratinocytes (HaCaTs) seeded on the nanofiber membranes were favorable. The collagen nanofibers were also found to lead to upregulation of involucrin, filaggrin, and TGase1 genes, indicating keratinocyte differentiation. *In vivo* placement of the collagen nanofibers in Sprague Dawley rat full-thickness skin wound models demonstrated an improvement in wound healing rate and reduced inflammation compared to Kaltostat and untreated controls. Other studies have primarily focused on the use of commercially-available collagen-based wound dressings such as Aplifraf, Dermagraft, Graftjacket, Integra, and others in human trials, for example for use in treating diabetic foot ulcers (Holmes et al., 2013).

Keratin, a protein that is a key component of skin, has been shown to aid in skin wound healing in multiple ways. Key factors that set keratin apart from other naturally-derived materials such as collagen include high homology between species to reduce immunogenicity of keratin-derived biomaterials, and a high degree of cystine presence to slow down degradation (Kelly, 2016). Cystine’s role as a precursor to glutathione, an antioxidant, provides a further mechanism by which keratin-based biomaterials can support healing (Kelly, 2016). Waters et al. (2018) evaluated the anti-inflammatory effects of various keratin-derived coatings *in vitro*. The wound healing and regenerative abilities of keratin-based biomaterials have been extensively studied, and Waters et al. (2018) demonstrated that peptide-containing keratin fractions with lower molecular weights appear to be slightly more effective in inducing a transition from pro-inflammatory macrophage phenotype to anti-inflammatory phenotype than high molecular weight keratin fractions.

Hyaluronic acid (HA) is a non-sulfated glycosaminoglycan and extracellular matrix component that acts as an immune regulator in physiological and pathological conditions (Noble et al., 2011). HA accumulates at sites of injury and inflammation where it is degraded by reactive oxygen species and hylauronidases, engaging in complex interactions with the wound environment to regulate cytokine secretion and influence immune cell migration (Voinchet et al., 2006). Methacrylation of HA enables light-activated crosslinking for easy formation of HA-based hydrogels (Ondeck and Engler, 2016). Chen et al. (2020) used methacrylated hyaluronic acid (Me-HA) hydrogels loaded with basic fibroblast growth factor (bFGF) as an injectable bioactive wound dressing (bFGF@Me-HA). When applied to mouse full-thickness skin wounds, bFGF@Me-HA improved wound healing with accelerated re-epithelialization, granulation formation, collagen deposition, and skin appendage regeneration. The hydrogels also led to improved cell proliferation and vascularization, which was caused by upregulation of transforming growth factor-β and VEGF.

Another naturally-derived biomaterial, soy protein isolate, has been shown to act as an anti-inflammatory agent by inhibiting NF- κB-dependent expression of inflammatory cytokines and VCAM-1 *in vivo* in acute and chronic inflammation models (Burris et al., 2014). Har-el et al. (2014) used soy protein isolate to electrospin soy protein scaffolds (SPS) for wound healing. When applied to a porcine full thickness excisional wound healing model, SPS treatment led to the presence of fewer inflammatory/immune cells than untreated controls and development of the beginnings of a stratified epithelial layer just 2 weeks after wounding. By 4 weeks after wounding, SPS treatment had led to dermal papillae formation in the dermis, collagen formation, and a well-formed stratified epithelial layer. The treatment also led to the formation of dermal appendages such as sweat glands and hair follicles, which were not observed in untreated controls.

### Combinatorial Biomaterials

Zhu et al. (2020) evaluated the anti-inflammatory effects of an injectable hydrogel composed of sodium alginate and bioactive glass (BG/SA hydrogel) that had previously been applied for skin tissue regeneration. They demonstrated that macrophages exposed to BG/SA hydrogels had enhanced gene expression of anti-inflammatory factors such as TGF-β, VEGF, bFGF, ARG and IL-10, and that cell culture media conditioned by BG/SA-treated macrophages led to increased migration of fibroblasts and mouse artery endothelial cells. Using a mouse full-thickness skin wound model in normal mice and macrophage-depleted mice, they found that the improvement in wound healing caused by BG/SA hydrogel was eliminated in the absence of macrophages, indicating the presence of macrophages played a direct role in *in vivo* wound healing with the BG/SA hydrogels.

A major concern with diabetic wounds is that they are persistently open due to slow healing, which can increase the potential for the development of infections. Acceleration of wound healing can reduce the opportunity for wound infection. In the context of diabetic wounds, even scarred healing would be an improved outcome compared to wound infection. PLGA is an FDA approved polymer that is biocompatible and biodegradable (Hirenkumar and Steven, 2012). While not commonly used by itself to induce immune effects, it is often used for applications in controlled drug delivery. Sun et al. (2021) sought to develop an immunomodulatory scaffold for diabetic wound management, mimicking the sequential appearance of fibrinogen and collagen I in the wound healing process. They developed coaxial scaffolds composed of PLGA nanofibers with fibrinogen incorporated into the shell and collagen I incorporated into the core, taking advantage of the PLGA degradation to expose collagen I over time. *In vitro*, the coaxial scaffolds were found to promote the secretion of growth factors associated with wound healing (TGF-b1, VEGF and bFGF), as well as immunosuppressive factors (COX-2 and TSG-6). Additionally, macrophages treated with media that had been conditioned by adipose-derived mesenchymal stromal cells (ASCs) exposed to the coaxial scaffolds had a greater M2/M1 ratio than those treated with PLGA scaffolds or PLGA scaffolds with only fibrinogen or only collagen I incorporated. When applied to an *in vivo* diabetic rat wound model, coaxial scaffolds were found to accelerate diabetic wound healing by resolving inflammation.

Materials based on methacrylic acid have shown great efficacy in treating various skin conditions, including diabetic wound healing (Fitzpatrick et al., 2012; Lisovsky and Sefton, 2016). Methacrylic acid biomaterials promote blood vessel formation *via* sonic hedgehog signaling and promote M2 macrophage polarization *via* an IGF-1 mediated pathway (Martin et al., 2010; Talior-Volodarsky et al., 2017). Plenty of theories as to how the methacrylic acid functions have been developed, but the full mechanism of action has not been fully detailed (Lisovsky et al., 2015).

The incorporation of cells or secretable factors involved in skin repair is another method by which biomaterials can be optimized for skin wound healing. In particular, stem cells are often used as immunomodulatory agents even if they do not develop into new tissue. The mechanisms behind immunomodulatory functions of stem cells are not entirely understood, but they likely result from a combination of soluble factor secretion and cell-to-cell interactions with immune cells (Kuo et al., 2012; Pumberger et al., 2016; Zhao et al., 2016). Chen et al. (2021) used cryogel/hydrogel biomaterials loaded with stem cells to generate an immunomodulatory response in a rat model of diabetic wound healing. The cell-seeded hydrogels/cryogels, which were formed from glycol chitosan and a biodegradable Schiff base crosslinker difunctional polyurethane, led to immunomodulation by down-regulating proinflammatory cytokines TNF-α and IL-1β and upregulating TGFβ-1. Chitosan, a cationic polysaccharide which is commonly derived from crustaceans, has been shown to have both pro-inflammatory and anti-inflammatory properties depending on conditions such as degree of deacetylation, molecular weight, form, and other factors (Fong and Hoemann, 2018). Anti-inflammatory effects of chitosan have been linked to intracellular signaling pathways involving cGAS-STING which can trigger a type 1 IFN response, inducing the release of anti-inflammatory factors such as IL-1ra (Fong and Hoemann, 2018). In the Chen et al. study, the combined effects of biomaterials and cells led wounds treated with cell-seeded biomaterials to also have a higher M2/M1 ratio than those of control wounds or wounds treated with stem cells alone, suggesting an anti-inflammatory function for the biomaterials. Uehara et al. (2019) developed anti-IL-6 antibody-eluting gelatin methacryloyl (GelMA) hydrogels to improve skin allograft survival (Uehara et al., 2019). GelMA is a commonly used biomaterial that is that is considered advantageous for its mechanical properties that can be precisely tuned. GelMA has been shown to exert anti-inflammatory properties by suppressing TNF-α expression (Donaldson et al., 2018). By crosslinking GelMA/anti-IL-6 on the wound bed prior to skin allograft placement, Uehara et al. (2019) enabled the continual release of anti-IL-6 at the wound site to combat IL-6, which plays a role in innate and adaptive immune responses. Implantation of GelMA/anti-IL-6 approach reduced T cell and monocyte infiltration into the allograft skin and almost doubled graft survival compared to the control. Importantly, incorporation of anti-IL-6 into the biomaterial also provided an improvement in graft survival compared to systemic administration of anti-IL-6.

Mao et al. (2019) aimed to address multiple phases of the skin healing process by developing a hydrogel with both anti-inflammatory and pro-angiogenic properties. They developed a polyethylene glycol (PEG) hydrogel that was crosslinked with silver ions (Ag^+^) and loaded with mangiferin liposomes (MF-Lip@PEG), with the goal of combining mangiferin’s cytoprotective properties with the antimicrobial activity of Ag^+^. When injected in a mouse skin flap injury model, the MF-Lip@PEG hydrogel was found to reduce tissue necrosis and increase microvessel density in the skin flaps. Furthermore, although PEG itself led to high macrophage recruitment, the MF-Lip@PEG hydrogel was able to reduce macrophage recruitment in a concentration-dependent manner.

In contrast to approaches that seek to dampen the immune response, Griffin et al. (2021) used the activation of specific immune responses by D-amino acid crosslinked Microporous annealed particle (MAP) hydrogels to elicit skin regeneration in murine models. They found that MAP hydrogels crosslinked with D-amino acids had faster degradation *in vivo* than those crosslinked with L-amino acids. This faster degradation was a result of enhanced immune cell recruitment, and was found to lead to tissue regeneration, in contrast to the formation of semi-fibrous dermal scar tissue when MAP hydrogels crosslinked with L-amino acids were used. These results suggest that the adaptive immune response can be exploited for skin wound healing.

## Conclusions and Further Directions

The fields of regenerative medicine and bioengineering have made great advances in developing biocompatible and regenerative scaffolds using biomaterials. As discussed, numerous biomaterials developed incorporate the immune system to naturally mediate regeneration and prevent scarring. Each of these approaches have their pros and cons, and it is likely that the best scaffold for regeneration will involve a combination of these materials. Understanding how these biomaterials promote regeneration *via* the immune system plays a crucial part in our understanding of how endogenous repair works. Countless papers highlight the role that various immune cells, particularly macrophages, play in mediating adult stem cell replication and differentiation into the target cell. By modulating the immune cell infiltrate into the injury site, it is possible to effectively modulate stem cell behavior. Hybrid biomaterials are a promising next step in regenerative medicine and will likely be able to combine the benefits of different materials. With hybrid materials come an exciting next step in understanding the healing process, creating more space for optimization. To achieve this goal, more research must be done on understanding all steps of the immune response to biomaterials, particularly *in vivo*.
